# Human REV3 DNA Polymerase Zeta Localizes to Mitochondria and Protects the Mitochondrial Genome

**DOI:** 10.1371/journal.pone.0140409

**Published:** 2015-10-13

**Authors:** Bhupendra Singh, Xiurong Li, Kjerstin M. Owens, Ayyasamy Vanniarajan, Ping Liang, Keshav K. Singh

**Affiliations:** 1 Department of Genetics, University of Alabama at Birmingham, Birmingham, Alabama, United States of America; 2 Department of Cancer Genetics, Roswell Park Cancer Institute, Buffalo, New York, United States of America; 3 Department of Biological Sciences, Brock University, St. Catharine’s, Ontario, Canada; 4 Departments of Genetics, Pathology, Environmental Health, Center for Free Radical Biology, Center for Aging and UAB Comprehensive Cancer Center, University of Alabama at Birmingham, Birmingham, Alabama, United States of America; 5 Birmingham Veterans Affairs Medical Center, Birmingham, Alabama, United States of America; National Institute of Environmental Health Sciences, UNITED STATES

## Abstract

To date, mitochondrial DNA polymerase γ (POLG) is the only polymerase known to be present in mammalian mitochondria. A dogma in the mitochondria field is that there is no other polymerase present in the mitochondria of mammalian cells. Here we demonstrate localization of REV3 DNA polymerase in the mammalian mitochondria. We demonstrate localization of REV3 in the mitochondria of mammalian tissue as well as cell lines. REV3 associates with POLG and mitochondrial DNA and protects the mitochondrial genome from DNA damage. Inactivation of *Rev3* leads to reduced mitochondrial membrane potential, reduced OXPHOS activity, and increased glucose consumption. Conversely, inhibition of the OXPHOS increases expression of *Rev3*. *Rev3* expression is increased in human primary breast tumors and breast cancer cell lines. Inactivation of *Rev3* decreases cell migration and invasion, and localization of *Rev3* in mitochondria increases survival and the invasive potential of cancer cells. Taken together, we demonstrate that REV3 functions in mammalian mitochondria and that mitochondrial REV3 is associated with the tumorigenic potential of cells.

## Introduction

Mitochondria are involved in energy metabolism, cell proliferation, cell growth, apoptosis, and other cellular regulatory mechanisms. Mitochondria contain their own DNA, which encodes 13 essential components of the respiratory chain and is replicated continuously in dividing cells and in postmitotic tissues. Failure to preserve the genetic integrity of the mitochondrial genome during replication results in depletion, deletion, or mutation of mitochondrial DNA (mtDNA), which impairs oxidative phosphorylation (OXPHOS) and causes cellular dysfunctions and diseases [1–4]. Mitochondrial DNA polymerase γ (POLG), the only polymerase described to date in human mitochondria, is the key protein involved in mtDNA replication and repair [5–7]. Mutations in *Polg1* are associated with depletion of mtDNA, mitochondrial malfunction, aging, carcinogenesis, and various other diseases [7,8]. However, the mechanisms by which mitochondria ensure stability and integrity of their own genome remain to be elucidated.

The capacity of a cell to duplicate its nuclear and mitochondrial genome in an error-free manner is necessary for maintaining homeostasis and limiting the risk for cancer and other diseases. The high fidelity of genome duplication is largely accredited to the proofreading capabilities of DNA polymerases delta and epsilon, which ensure that a correct nucleotide is incorporated at each step during DNA replication. But when these polymerases encounter an altered template due to covalent adducts and/or distortions in the secondary structure of DNA, the fidelity and processivity become limiting, and the replication fork stalls due to inability of these polymerases to accommodate bulky lesions or deformed structures within the DNA template. To overcome these conditions, cells have evolved a mechanism to promote replication despite such an altered template, a process known as translesion DNA synthesis (TLS). TLS bypasses the lesion and avoids the stall in the replication fork. After such adjustment to damage, the DNA can be repaired by the cell.

TLS is mediated by specialized DNA polymerases [9], including DNA polymerase zeta (Pol zeta, catalytic subunit REV3L). As a TLS polymerase, Pol zeta lacks the characteristic proofreading activity present in other B-family DNA polymerases [10]. Its two main subunits are REV3L, the catalytic subunit, and REV7, the structural subunit. Although REV3L alone is capable of polymerization, association of REV3L and REV7 is associated with stabilization of Pol zeta [11].

The human *Rev3L* gene encodes a ~350 kDa protein (REV3L) containing a large C-terminal DNA polymerase subunit. Human *Rev3L* (hereafter *Rev3*) is ubiquitously expressed in tissues and is involved in the bypass of many types of DNA damage induced by ultraviolet (UV) radiation [12–14] and chemical damaging agents [13]. Deletion of *Rev3* is embryonically lethal [15,16], whereas over-expression of *Rev3* leads to increased spontaneous mutations [17]. *Rev3* is required for efficient replication of the common fragile site during the G2/M phase, and the resulting fragile site instability in *Rev3* knockout mice may be associated with cell death during embryonic development [18]. Spontaneous chromosomal instability is present in *Rev3*-deleted mouse fibroblasts and *Rev3*-deleted cell lines [19,20]. Recent reports support the essential role of *Rev3* in proliferation of normal mammalian cells [12,21]. Although expression of REV3 is necessary for normal physiology of cells, over-expression of REV3 is associated with breast cancers and brain gliomas [22,23]. Further, depletion of *Rev3* sensitizes mouse B-cell lymphomas, lung adenocarcinoma, and human brain gliomas to cisplatin [23–25].

We have earlier reported that REV3 localizes to mitochondria in *Saccharomyces cerevisiae* yeast cells and participate in mtDNA mutagenesis [26]. In this report, we provide evidence that human REV3 also localizes to mitochondria and that inactivation of *Rev3* leads to mitochondrial dysfunction.

## Materials and Methods

### Construction of expression vectors for REV3 localization and confocal microscopy

The REV3 mitochondrial localization signal (MLS) and nuclear localization signal (NLS) were predicted by use of MitoProt II and PredictNLS Online software, respectively. The Rev3MLS and Rev3NLS fragments were produced by PCR on pcDNA3.1+Flag, which contained a full-length *Rev3* gene (a gift from Dr. Yoshiki Murakumo). The PCR products were cloned into a pEGFP-N2 plasmid (Clontech, Palo Alto, CA). The primers for Rev3MLS were Xho1-5’-GATCTCGAGGATGGTTATGGACAGCAGCC and BamH1-5’-GGTGGATCCCTGCTTTTCGGAACTTGACAGC, and, for Rev3NLS, were Xho1- 5’-CAGATCTCGAGATCCCATGGAAATTGGTGAA and BamH1-5’-CGGTGGATCCTAACCTCAGCA CCAGACTGAGA. Transfection of NIH 3T3 cells was accomplished with Fugene HD (Roche, Indianapolis, IN). Similarly, the full length Rev3-pcDNA3.1+Flag vector was transfected in HEK293 cells (HEK293 cells have very low level of endogenous REV3 expression) using Fugene HD reagent (Roche). Full length REV3 expressing cells were used for immunodetection of REV3 using antiFlag-M2 antibody (Sigma-Aldrich, St. Louis, MO). Cell staining was performed with Mitotracker (Molecular Probes, Eugene, OR) and 4', 6-diamidino-2-phenylindole (DAPI, Vector Laboratories, Burlingame, CA). Confocal pictures were taken with a Leica confocal instrument. Analysis of the pictures was carried out with ImageJ software (NIH, http://rsbweb.nih.gov/ij/).

### Construction of expression vectors containing mutant Rev3 cDNA

A *Rev3* mutant was constructed by removing, by use of XbaI, a 3.6-kb fragment that contained the NLS from the full-length *Rev3*. This mutant was named pcDNA3.1+Flag Rev3+MLS-NLS (Rev3 with MLS but without NLS). A second mutant, pcDNA3.1+Flag Rev3-MLS+NLS (Rev3 with NLS but without MLS), was designed to remove the MLS. The full-length Rev3 plasmid was digested with EcoRI and AleI, and a 1.75-kb fragment including the MLS was removed. PCR was used to produce a 1.225-kb fragment without the MLS but with EcoRI and AleI at the 5’ and the 3’ ends. The PCR product was placed into the backbone of the digested Rev3 plasmid so that a 0.425-kb fragment with the MLS was knocked out. The Rev3+MLS-NLS and the full-length Rev3 were transfected into HEK293 cells, and stable clones were selected.

### Gene expression analyses

Rev3^+/+^ and Rev3^-/-^ cells were used for analysis of expression of *Polg1*, *Polg2* and *Cox II* genes. Rev3^+/+^ cells exposed to 5 J/m^2^ of UV were used for *Rev3* gene expression studies. Expression of *Rev3* was analyzed in tetracycline inducible (Tet-on) human mammary epithelial breast cancer cell line MCF7 (MCF7) and Tet-On MCF7 Polg1 D1135A (MCF7 Polg1DN) cells after 12 days of induction with Doxycycline (1 μg/ml). Tet-On MCF7 Polg1DN cells were prepared as reported earlier [7]. Expression of Polg1 D1135A, a dominant negative mutation, leads to depletion of mitochondrial DNA [7]. Total RNA was isolated and reverse-transcribed using standard procedures. RT PCR was used to measure the expression levels of these genes. Beta-2-microglobulin (B2M) or β-actin was used as an internal control.

### Rev3 knockdown by shRNA

The *Rev3* shRNA was synthesized as 5’-TAGTAGTCTGCAGTCACTATCCTTACTGGAAGCTTGCG GTGAGGATAGTGACTGCGGACTATTACATTTTTTT-3’ [27] in the pGPU6/GFP/Neo vector (GenePharma, Shanghai). The *Rev3* shRNA and its control shRNA were transfected into HeLa cells using Fugene HD (Roche), and stable clones were selected. The RNA samples were extracted with Trizol (Invitrogen, Carlsbad, CA). cDNA synthesis was accomplished with SuperSctrip III kits (Invitrogen). Real-time PCR using SYBR Green (Invitrogen) was employed to identify *Rev3* knockdown in the clones.

### Mitochondrial membrane potential measurement

Mitochondrial membrane potential was measured by the fluorescence of tetramethylrhodamine ethyl ester (TMRE) (Molecular Probes). Cells were incubated with 100 nM TMRE for 35 min, harvested, and suspended in PBS. The fluorescence of the cells was read on the FL2 channel of a Becton Dickinson FACScan Flow Cytometer (Franklin Lakes, NJ).

### ROS measurements

Oxidation of dihydroethidium (DHE) (Molecular Probes) fluorescent probe was used to measure intracellular ROS. Cells were labeled with 10 μg/ml DHE for 40 min, harvested, and suspended in PBS. The fluorescence of the cells was read on the FL2 or FL1 channel of a Becton Dickinson FACScan Flow Cytometer.

### Mitochondrial OXPHOS enzyme activities

Mitochondrial OXPHOS enzyme activities were measured on enhanced mitochondrial preparations as previously described [28,29]. Complex I activity was measured as the rate of NADH oxidation in 25 mM potassium phosphate (pH 7.2), 5 mM MgCl_2_, 2.5 mg/ml BSA, 65 μM coenzyme Q1, 2 μg/ml antimycin A, and 2 mM KCN. The rate of absorbance change at 340 nm with a 425 nm reference wavelength was measured for 2 min. To measure complex II activity, total cellular protein was stimulated with and without 20 mM succinate for 10 min at 30°C in 25 mM potassium phosphate (pH 7.2), 5 mM MgCl_2_, 2.5 mg/ml BSA, and 2 mM KCN. Coenzyme Q_1_ (65 μM), antimycin A (2 μg/ml), rotenone (2 μg/ml), and 2, 6-dichlorophenolindophenol (DCIP, 50 μM) were added to the reaction system, and the rate of reduction of DCIP was measured at 600 nm for 3 min. Complex III activity was measured with cytochrome c (III) (15 μM) in 25 mM potassium phosphate (pH 7.2), 5 mM MgCl_2_, 2.5 mg/ml BSA, 2 mM KCN, 2 μg/ml rotenone, 0.5 mM n-dodecyl β-maltoside, and 35 μM coenzyme Q_2_H_2_. The rate of absorbance change at 550 nm with a 580 nm reference wavelength was read for 2 min. Complex IV activity was measured by the oxidation of cytochrome c (II) (15 μM) in 20 mM potassium phosphate (pH 7.0) and 0.5 mM n-dodecyl β-maltoside. The rate of absorbance change at 550 nm with a 580 nm reference wavelength was measured for 2 min.

### Western blot analyses

Cells were lysed in RIPA lysis buffer (50 mM Tris, pH 7.4; 150 mM NaCl; 1 mM PMSF; 1 mM EDTA; 1% Triton x-100; 1% sodium deoxycholate; and 0.1% SDS) with addition of Protease Inhibitor Cocktail (Thermo Fisher Scientific, Waltham, MA) and Phosphatase Inhibitor Cocktail I (Sigma-Aldrich). Nuclear, cytoplasmic and mitochondrial fractions were prepared as described earlier [30]. Fifteen to thirty microgram protein was size fractionated on a 12% sodium dodecyl sulfate—polyacrylamide (SDS-PAGE) gel (7.5% SDS-PAGE was used for REV3 western blot), and transferred onto a polyvinylidene difluoride membrane (Millipore Corp., Billerica, MA) using the wet transfer system and blocked in 5% skim milk in PBST for 1 h. A premixed cocktail (7.2 μg/ml) containing primary monoclonal antibodies against subunits of OXPHOS complexes (Mitosciences, Eugene, OR) was used to detect representative subunits from OXPHOS complex I, II, III and V. For detection of OXPHOS complex IV subunit COX II, an antibody from Life Technologies (Grand Island, NY) was used. Rabbit polyclonal primary antibodies against POLG1 and POLG2 (gifts from Dr. William C. Copeland, NIEHS) and Pol zeta (REV3L, Santa Cruz Biotechnology, Santa Cruz, CA, Cat# sc-48814) were used to detect the expression of these proteins. α-Tubulin, Lamin B1, and Tom20 (Santa Cruz Biotechnology) antibodies were used as markers for cytoplasmic, nuclear, and mitochondrial protein fractions, respectively. HRP-conjugated secondary antibodies (Vector Laboratories) and ECL reagent kits (GE Healthcare Biosciences, Pittsburg, PA) were used for film development.

### Glucose consumption

Rev3^+/+^ and Rev3^-/-^ cells were plated in 6-well dishes and, at 24 h after seeding, fresh growth media was added. Every subsequent 24 h, the total cell numbers were counted with a hemocytometer. Glucose in media samples was measured with a OneTouch Ultra LifeScan Gluocometer (Milpitas, CA). Glucose consumption was calculated as (glucose in fresh media—glucose in media sample)/cells per well.

### Cell survival assay

HEK293 cells containing full-length *Rev3*, Rev3+MLS-NLS, or a control vector clone were seeded into 60-mm dishes and cultured overnight. The cells were treated with 7.5 J/m^2^ of ultraviolet (UV) light. The cells were trypsinized and then seeded into six-well plates at 2000 cells per well. The control cells were seeded at 500 cells per well. The cells were cultured for 10 days and then fixed with methanol. The colonies were stained with 0.01% coomassie blue (in 10% methanol and 10% acetic acid) for 15 min. Cell colonies with at least 50 cells were counted under a stereo microscope, and the surviving fractions were calculated.

### Cell-culture migration assay

Cell-culture migration assay was carried out as described earlier [31]. Briefly, stable HeLa cell clones of *Rev3* shRNA or control shRNA, seeded into six-well plates, reached confluence on the next day. The cells were wounded with yellow pipet tips. Pictures of the wounded lines at the same positions were taken at 0 and 72 h after wounding.

### Matrigel invasion assay

The Matrigel invasion assay was accomplished with BD BioCoat Matrigel Invasion Chambers (Cat. 354480, BD Biosciences, San Jose, CA). HEK293 stable clones of *Rev3* were seeded as 1x10^5^ cells per chamber; HeLa stable clones of *Rev3* shRNA or the control shRNA were seeded as 0.5 x10^5^ cells per chamber. DMEM media with 10% FBS was used as the chemoattractant. Cells were allowed to migrate for 24 h, and then the membranes were stained with the Diff-Quik Stain Set (Dade Behring, Newark, DE). The invading cells were counted in 6 views per membrane under a microscope at 20X magnification. Cell counts were averaged and statistically analyzed.

### Mitochondrial DNA damage and mtDNA content analysis

To induce mtDNA damage, Rev3^+/+^ and Rev3^-/-^ mouse embryonic fibroblast cells were treated with 2.5 or 5 J/m^2^ of UV radiation by a Stratalinker UV Crosslinker 2400 (Stratagene, La Jolla, CA). UV-irradiated cells were harvested at 6 and 24 h post-treatment, and their DNA was isolated with QIAamp DNA mini kits (Qiagen, Valencia, CA). The mitochondrial DNA content was analyzed by real-time PCR by absolute quantification with the following primers: mMitoF: 5'-CTAGAAACCCCGAAACCAAA-3', mMitoR: 5'-CCAGCTATCACCAAGCTC GT-3', mB2MF: 5'-ATGGGAAGCCGAACATACTG-3', and mB2MR: 5'-CAGTCTCAGTGGGGGTG AAT -3'. B2M was used as an internal control.

For analysis of mtDNA damage, HEK293 cells containing full-length *Rev3*, Rev3-MLS, or control vector were treated with 2.5 J/m^2^ of UV light and cultured for 6 h, and DNA was isolated from irradiated cells by use of QIAamp DNA mini kits (Qiagen). mtDNA damage was assessed by amplifying long (L) and short (S) fragments of mtDNA with the following primers: L-F: 5'-CACACGAGAAAACACCCTCA-3', L-R: 5'-CTATGGCTGAGGGGAGTCAG-3', S-F: 5'-TCCAACTCATGAGACCCACA-3', and S-R: 5'-TGAGGCTTGGATTAGCGTTT-3', as described earlier [32].

### RT PCR after treatment of cells with mitochondrial inhibitors

143B parental cells were treated with the mitochondrial enzyme complex inhibitors rotenone (20 μM), TTFA (25 μM), antimycin (10 μM), and KCN (10 mM) for 2 h, which block complex I, II-UQ, III, and IV, respectively. The cells were collected and total RNA was isolated, and cDNA was synthesized by following standard procedures. RT PCR was performed to analyze the expression of *Rev3*.

### Chromatin immunoprecipitation (ChIP) assays

ChIP assays were performed with Rev3^+/+^ and Rev3^-/-^ cells using SimpleChip Enzymatic Chromatin IP Kits (Cell Signaling, Danvers, MA), as suggested by the manufacturer. In brief, Rev3^+/+^ and Rev3^-/-^ cells (~5 x10^8^), grown in 100-mm tissue culture dishes, were cross-linked with 1% formaldehyde, digested with micrococcal nuclease, and sonicated. Soluble chromatin was collected and incubated overnight at 4°C on a rotating platform with a antibody against DNA polymerase zeta (gift from Dr. Christopher W. Lawrence, University of Rochester, and Santa Cruz Biotechnology), which detects REV3. The DNA was recovered and subjected to real-time PCR analysis with mouse D-loop and Cox II primers. These primers, used for end point real-time PCR amplification by the SYBR green method (Bio-Rad Laboratories, Hercules, CA), were as follows: D-Loop forward primer, 5'-CAGTGGCATGCACCCAGGGAA-3', and reverse primer, 5'-GCATGC CCCTTTTAGCCTTGGCA-3'; Cox II forward primer 5'-CAGTGGCATGCAC CCAGGGAA-3', and reverse primer 5'-GCATGCCCCTTTTAGCCTTGGCA-3'. Amplification of chromatin before immunoprecipitation at a dilution of 1:50 was used as a positive control (input); ChIP with rabbit serum IgG served as a negative control. The assays were accomplished in three replicates. Agarose gel electrophoresis of PCR-amplified products for ChIP DNA and input DNA samples were used to represent the results.

### Co-immunoprecipitation (Co-IP) assay

Co-IP assays were accomplished with Rev3^+/+^ and 143B cells with or without UV exposure. Co-IP with Rev3^-/-^ cells were used as negative control. In brief, cells in 100-mm tissue culture dishes were grown for 6 h after treatment with 5 J/m^2^ of UV light. Untreated cells were used as controls. For immunoprecipitation, cells were lysed for 8 min on ice with lysis buffer (20 mM Tris-HCl, pH 8; 150 mM NaCl; 0.1% NP-40; and 1X Protease Inhibitor (Thermo Fisher Scientific, Waltham, MA) and the lysate was centrifuged at 4800 rpm for 10 min. Supernatant protein contents were measured with protein assay kits (Bio-Rad Laboratories). Supernatant (250 μg protein) was pre-cleared with Magnetic Protein A Dynabeads (Invitrogen; 35 μL beads/mL lysate) for 1 h and then used for Co-IP with 10 μg of Pol zeta primary antibody. The lysate was incubated with antibody overnight on a rotator at 4°C. Magnetic Protein A Dynabeads were added, and the preparations were incubated for 1 h on a rotator at 4°C. Beads were washed in lysis buffer (five times) and captured with a magnetic separator (Qiagen). Proteins were eluted in SDS sample buffer at 65°C for 10 min and with frequent vortexing. The protein samples were separated by SDS-PAGE on a 10% polyacrylamide gel and electroblotted onto a PVDF membrane. The blots were incubated with specific primary rabbit polyclonal antibodies against POLG1 and POLG2 (gift from Dr. William C. Copeland, NIEHS) and detected with an HRP-conjugated secondary antibody (Vector Laboratories). Generic ECL reagent kits (GE Healthcare Biosciences) were used for film development.

### Rev3 expression in breast tumors

All experiments were approved by the Roswell Park Cancer Institute Institutional Review Board, permit number I92106. Consent from patients was not needed, as anonymous tissue samples were used for study. Normal and matched breast tumor RNA samples were obtained from the biorepository resource facility of the Roswell Park Cancer Institute and provided to us under IRB-approved permit number I92106. RNA (1 μg) was reverse transcribed, and the gene expression of *Rev3* was analyzed by RT PCR.

### Statistical analyses

All statistical analyses were performed with Sigma Plot 11.0 software (Systat Software, San Jose, CA). Data were compared using two-tailed Student's t-tests.

## Results

### Human REV3 localizes to mitochondria

**Fig 1:**
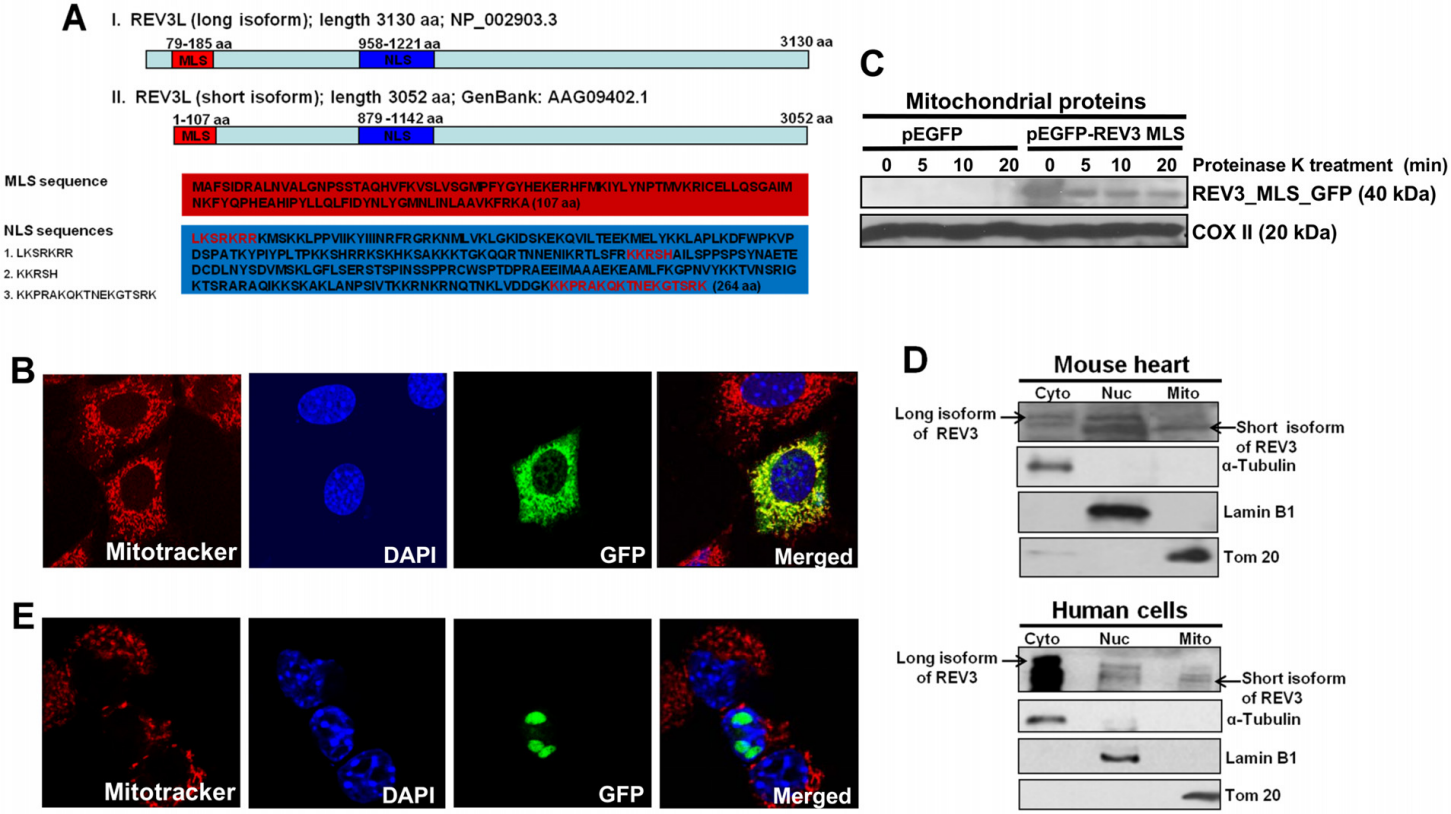
Human REV3L contains a mitochondrial localization signal (MLS) and localizes to mitochondria. (**A**) Schematic drawings of the MLS and NLS in the long and short forms of human REV3L. Amino acid numbers and sequences of MLS and NLS are indicated. (**B**) Fluorescence microscopic pictures showing the localization of Rev3MLS attached GFP protein in mitochondria of NIH3T3 cells. (**C**) Western blot analysis showing mitochondrial localization of Rev3MLS attached GFP protein and mitochondria-encoded COX II protein using protein extract from proteinase K-treated mitochondria. (**D**) Western blot analyses of cytoplasmic (cyto), nuclear (nuc), and mitochondrial (mito) protein fractions from mouse heart and human breast epithelial cell line MDA-MB-231 showing the localization of long and short isoforms of REV3 protein in different cellular compartments. (**E**) Fluorescence microscopic pictures showing the localization of Rev3NLS attached GFP protein in the nucleus of NIH3T3 cells.

In our previous study conducted with yeast, we demonstrated that REV3 functions in mitochondria [26]. Using MitoProt II software, we have now identified, at the N-terminal region of the human REV3 protein, a mitochondrial localization signal (MLS) that facilitates translocation of this protein to mitochondria. We analyzed two known isoforms of human REV3 synthesized from the same *Rev3* gene via the use of two alternative translation initiation (ATI) sites. Human *Rev3* mRNA contains two AUGs. Translation initiation from the first AUG synthesizes a long isoform of REV3 (3130 amino acid; ~352kDa; NP_002903.3), and initiation from the second AUG generates a small isoform of REV3 (3052 amino acid; ~343kDa; AAG09402.1). The import of proteins into the mitochondria is frequently dependent on an N-terminal, positively charged amphipathic α-helix, which functions as an MLS [33]. Our analyses for MLS sequence using MitoProt II revealed that short isoform of REV3 contains a 107-amino acid long putative MLS sequence at the N-terminus of the protein (76.9% confidence, MitoProt II). The long isoform of the REV3 protein contains an additional 78 amino acid sequence at the N-terminus (Fig 1A).

On the basis of this prediction, the corresponding nucleotide sequence for these 107-amino acids, Rev3MLS was produced by PCR. The PCR product was cloned into a pEGFP-N2 plasmid and transfected into NIH3T3 cells. Mitotracker and DAPI dyes were used to locate the mitochondrial and nuclear compartments, respectively (Fig 1B). Merged images show the localization of passenger protein GFP in mitochondria. These analyses suggest that the N-terminus 107-amino acid sequence of short isoform of REV3 contains an active mitochondrial localization signal that has the ability to direct localization of human REV3 into mitochondria. Full length protein expression of REV3 in mitochondria was also detected by immunofluorescence in Rev3-pcDNA3.1Flag transfected HEK239 cells using anti-Flag-M2 antibody (S1 Fig).

We performed Western blot analyses of extracts prepared from proteinase K (0.014%)-treated mitochondria isolated from HEK293 cells (HEK293 cells have very low level of endogenous REV3 expression) transfected with pEGFP-N2 vector or pEGFP-N2-Rev3MLS expressing the fusion protein. Fig 1C shows a single band of ~40 kDa REV3_MLS_GFP protein only in cells transfected with the Rev3MLS construct. Proteinase K treatment confirmed that Rev3MLS localizes the passenger protein GFP inside the mitochondria, not to the mitochondrial outer membrane. The membrane was stripped and re-probed with antibody against COX II antibody (20 kDa), an authentic mitochondrial protein (Fig 1C). To further confirm the localization of REV3 in mitochondria, we performed Western blots with cytoplasmic, nuclear, and mitochondrial protein fractions from mouse primary organ heart as well as from the human breast epithelial cell line, MDA-MB-231. We detected presence of both long and short isoforms of REV3 in cytoplasm and nuclear fractions while only short isoform of REV3 localized to mitochondria in both mouse and human samples (Fig 1D). Specificity of the REV3 antibody was confirmed by Western blot with protein samples from Rev3^+/+^ and Rev3^-/-^ cells and Rev3 shRNA transfected HeLa cells (S2 Fig). The presence of full-length REV3 protein in mitochondria indicates towards a possible role of this DNA polymerase in mitochondria.

Analyses performed with PredictNLS software predicted the presence of a nuclear localization signal (NLS) in both isoforms of human REV3 (Fig 1A), and we confirmed the capacity of this predicted NLS region for translocation of GFP to nucleus (Fig 1E).

### Rev3 inactivation inhibits mitochondrial functions

The above observations suggest that REV3 is localized to mitochondria. Since OXPHOS is the major metabolic pathway in mitochondria, whether the presence of REV3 in mitochondria is indispensable for mitochondrial functions was assessed. Rev3^+/+^ and Rev3^-/-^ mouse embryonic fibroblasts (MEFs) (gifts from Dr. Richard D. Wood) were used to measure endogenous expression levels of mitochondrial genome-encoded gene *Cox II*. In these cells, changes in enzymatic activities and protein expression of OXPHOS complexes I-V in the presence or absence of REV3 were measured, and the effect of inactivation of *Rev3* on mitochondrial membrane potential and intracellular ROS levels was examined.

**Fig 2:**
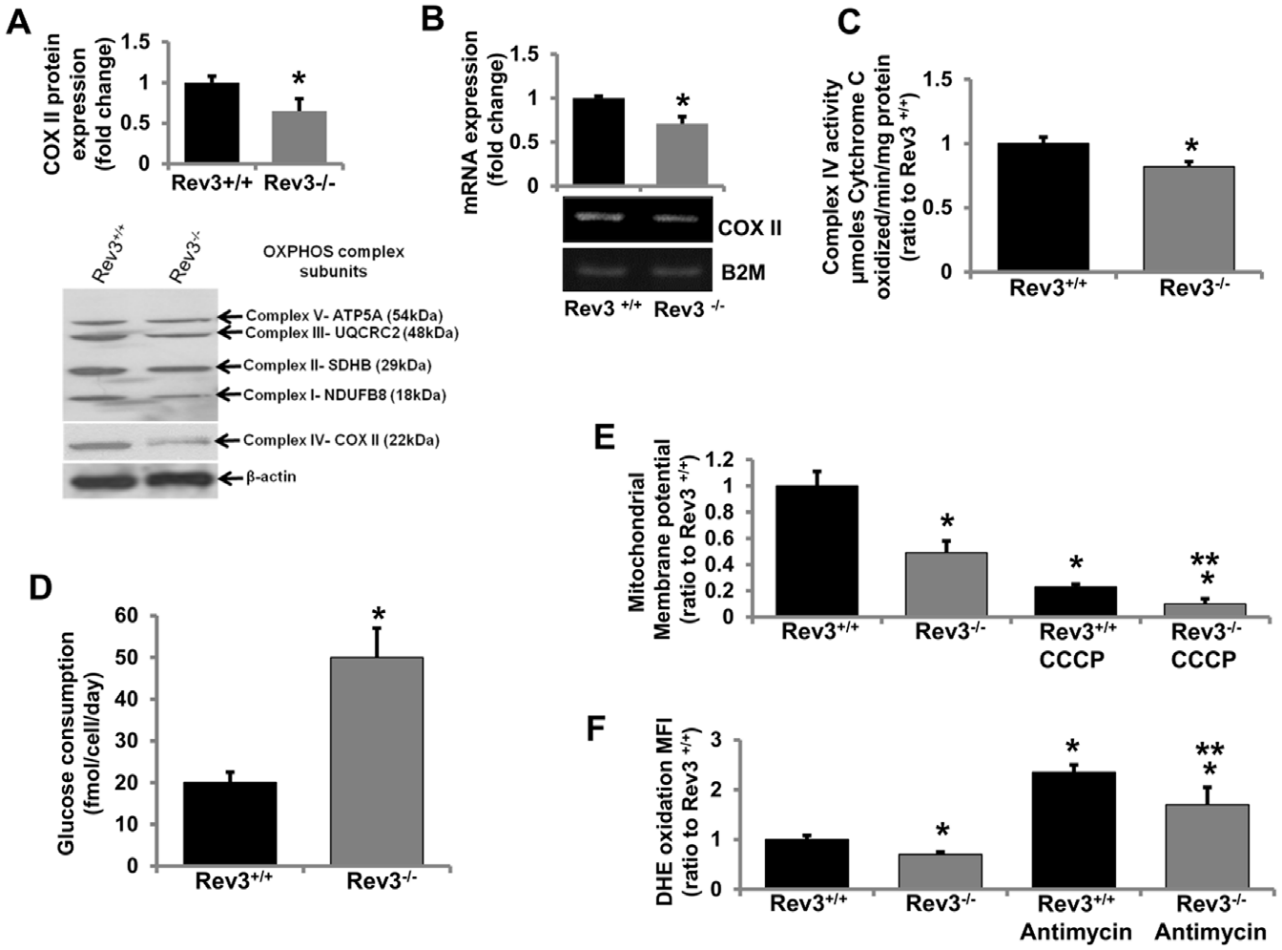
*Rev3* inactivation affects mitochondrial functions. (**A**) Western blot analysis of representative subunits of mitochondrial respiratory complexes I-V in Rev3^+/+^ and Rev3^-/-^ mouse embryonic fibroblasts. The bar graph represents fold change in COX II protein expression (mean ± s.d.) from at least three independent experiments. *P<0.05 versus Rev3^+/+^; Student's t-test. (**B**) RT PCR analyses of mitochondrial-encoded gene *Cox II* in Rev3^+/+^ and Rev 3^-/-^ cells. The bar graph represents fold change in mRNA expression (mean ± s.d.) from at least three independent experiments. *P<0.05 versus Rev3^+/+^; Student's t-test. (**C**) Enzymatic activity of mitochondrial respiratory complex IV in Rev3^+/+^ and Rev3^-/-^ cells. Bars represent the means ± s.d. *P<0.05 versus Rev3^+/+^; Student's t-test. (**D**) Glucose consumption by Rev3^+/+^ and Rev3^-/-^ cells. Bars represent the mean ± s.d. *P<0.05 versus Rev3^+/+^; Student's t-test. (**E**) Mitochondrial membrane potential of Rev3^-/-^ cells relative to Rev3^+/+^ cells in the presence or absence of a mitochondrial membrane potential inhibitor CCCP treatment. Bars represent the mean ± s.d. *P<0.05 versus Rev3^+/+^ and **P<0.05 versus Rev3^+/+^ CCCP; Student's t-test. (**F**) ROS production in Rev3^+/+^ and Rev3^-/-^ cells in the presence or absence of OXPHOS inhibitor Antimycin A treatment. Bars represent the mean ± s.d. *P<0.05 versus Rev3^+/+^ and **P<0.05 versus Rev3^+/+^ Antimycin A; Student's t-test.

For OXPHOS complex IV, which has three mitochondrial-encoded proteins, lower expression of COX II, a mitochondrial-encoded complex IV protein (Fig 2A), lower enzymatic activity (Fig 2C), and the lower expression of mitochondrial genome-encoded gene *Cox II* (Fig 2B) in Rev3^-/-^ cells relative to Rev3^+/+^ cells indicated a role of REV3 in regulation of mitochondrial genes/genome and OXPHOS functions. There were no significant changes in the activities of other complexes of mitochondrial OXPHOS in Rev3^-/-^ cells relative to Rev3^+/+^ cells (data not shown).

Since mitochondrial OXPHOS is the main source for production of ATP, cells with compromised OXPHOS switch their metabolism towards glycolysis [34]. Therefore, the glucose consumption was measured in Rev3^+/+^ and Rev3^-/-^ MEFs. The higher glucose consumption rate in Rev3^-/-^ cells (Fig 2D) indicates a compromised OXPHOS system and altered mitochondrial metabolism in these cells. Mitochondrial membrane potential, an indicator of the capacity of the cells to pump hydrogen ions across the inner membrane during energy production by OXPHOS, was also decreased in Rev3^-/-^ cells (Fig 2E). Treatment with carbonyl cyanide m-chlorophenyl hydrazine (CCCP), an inhibitor of OXPHOS, decreased the membrane potential in Rev3^-/-^ cells relative to Rev3^+/+^ cells (Fig 2E). Mitochondria produce reactive oxygen species (ROS) during oxidative phosphorylation [29]. ROS production in the form of superoxide radicals was low in Rev3^-/-^ cells (Fig 2F). Further, after treatment with the mitochondrial inhibitor antimycin A, there was less ROS production in Rev3^-/-^ cells relative to Rev3^+/+^ cells (Fig 2F).

### OXPHOS inhibition increases Rev3 expression

**Fig 3:**
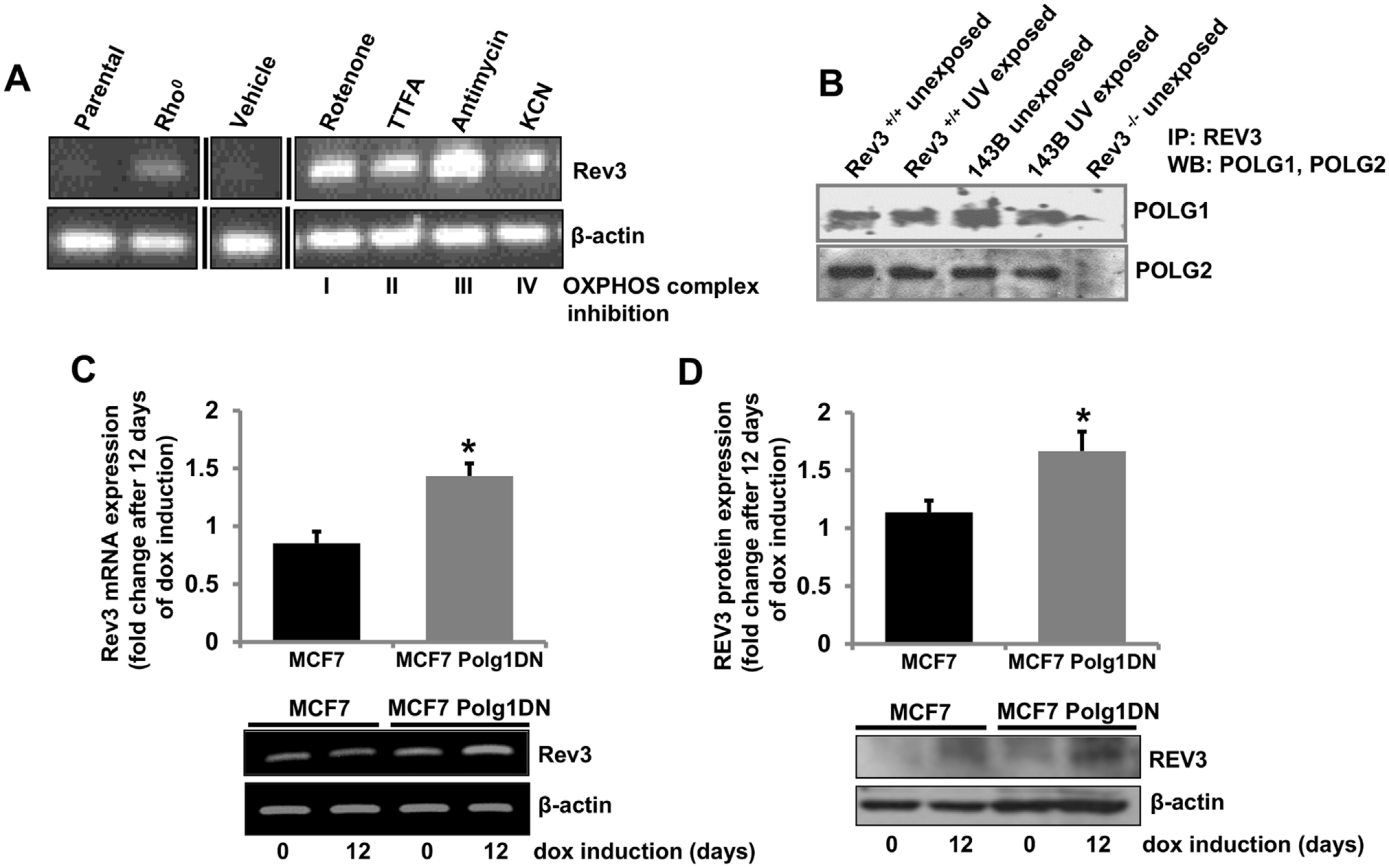
Inhibition of OXPHOS increases REV3 expression. (**A**) RT PCR analysis of *Rev3* gene expression in 143B parental and rho° cells. Treatment of 143B parental cells with mitochondrial OXPHOS inhibitors increases expression of *Rev3*. Black vertical lines indicate where intervening lanes have been removed for clarity. (**B**) Co-immunoprecipitation of POLG1 and POLG2 with REV3 in Rev3^+/+^ and 143B cells with or without UV treatment. Rev3^-/-^ cells were used as negative control. (**C**-**D**) RT PCR (**C**) and Western blot (**D**) analyses showing increased mRNA and protein expression of REV3 in MCF7 cells containing tetracycline-inducible, dominant negative mutant POLG1 (MCF7 Polg1 DN) after 12 days of dox induction. The bar graphs represent fold change in mRNA or protein expression (mean ± s.d.) from at least three independent experiments. *P<0.05 versus MCF7 cells at day 0; Student's t-test.

Since *Rev3* inactivation altered mitochondrial functions, we examined whether mitochondrial dysfunction similarly affects expression of the *Rev3* gene. For this, mRNA expression of *Rev3* in rho° cells (mtDNA deficient) was measured and compared with *Rev3* expression in parental cells (Fig 3A). mRNA expression of *Rev3* was higher in rho° cells relative to parental cells (Fig 3A). Increased expression of *Rev3* in rho° cells suggests a role of *Rev3* in metabolism of mtDNA (Fig 3A). Similarly, *Rev3* expression was also assessed in cells in which OXPHOS was inhibited by specific inhibitors of the OXPHOS complexes (Fig 3A). There was increase in *Rev3* mRNA expression after treatment with each inhibitor (Fig 3A).

### REV3 associates with mitochondrial DNA Polymerase γ

We show localization of human REV3 in mitochondria and DNA polymerase γ is known to be present in mitochondria [35,36]. We determined, by immunoprecipitation experiments, if REV3 associates with mitochondrial polymerase γ in mitochondria. This was accomplished with protein samples from Rev3^+/+^ and 143B cells exposed or unexposed to UV (Fig 3B). Positive Western blots with both subunits (POLG1 and POLG2) of polymerase γ with precipitated protein samples from both unexposed and UV-exposed Rev3^+/+^ and 143B cells using REV antibody suggests an association of REV3 with polymerase γ in mitochondria (Fig 3B).

Since REV3 associates with polymerase γ, we determined if inhibition of polymerase γ affects expression of *Rev3* by use of cells expressing the tetracycline-inducible, dominant negative mutant of POLG1 (Polg1DN) described earlier [7]. Earlier, we have shown depletion of mtDNA content in MCF-7 cells by induction of this dominant negative mutant of POLG1 [7]. After induction of the dominant negative POLG1 with doxycycline (dox, 1 μg/ml), there was induction of *Rev3* mRNA and protein expression (Fig 3C and 3D).

### REV3 protects cells from mtDNA damage

**Fig 4:**
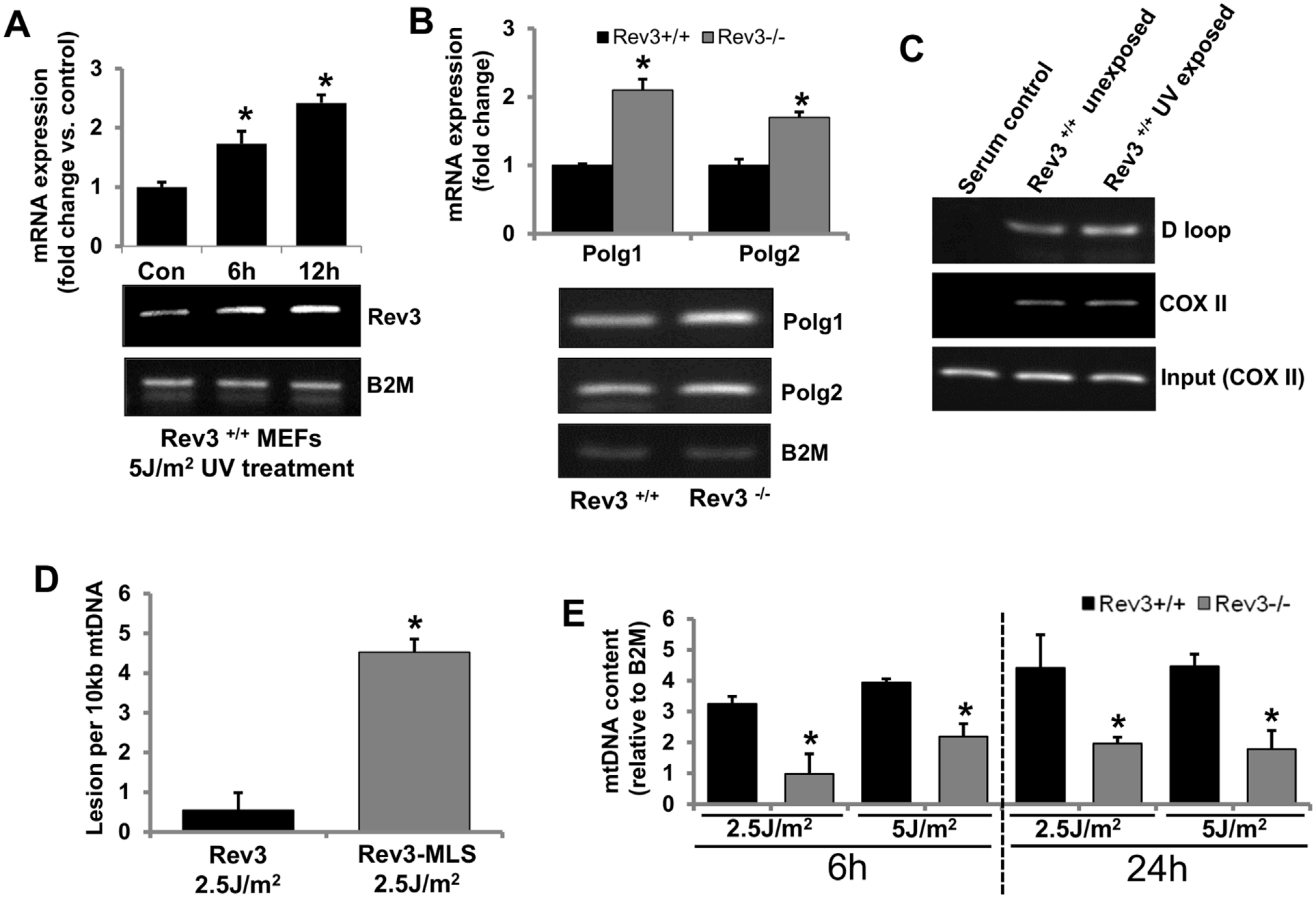
REV3 protects cells from mtDNA damage. (**A**) RT PCR results showing a time-dependent increase in *Rev3* gene expression in Rev3^+/+^ cells after UV exposure. The bar graph represents fold change in mRNA expression (mean ± s.d.) from at least three independent experiments. *P<0.05 versus UV-unexposed control; Student's t-test. (**B**) RT PCR analysis showing increased expression of *Polg1* and *Polg2* in Rev3^-/-^ cells relative to Rev3^+/+^ cells. The bar graph represents fold change in mRNA expression (mean ± s.d.) from at least three independent experiments. *P<0.05 versus Rev3^+/+^; Student's t-test. (**C**) Representative ChIP results showing binding of REV3 to the mtDNA (D loop and COX II region) in Rev3^+/+^ cells. Experiment was carried out in triplicate. A further increase in REV3 binding to the D loop region is evident after UV exposure. (**D**) mtDNA damage analysis in UV-exposed (2.5 J/m^2^) HEK293 cells expressing full-length *Rev3* and HEK293 cells expressing *Rev3* without the MLS (Rev3-MLS) after 6 h of exposure. Bars represent the mean ± s.d. *P<0.05 versus full-length Rev3 expressing cells; Student's t-test. (**E**) mtDNA content of UV-exposed (2.5 and 5 J/m^2^) Rev3^+/+^ and Rev3^-/-^ cells after 6 and 24 h of exposure. Bars represent the mean ± s.d. *P<0.05 versus respective UV-unexposed cells; Student's t-test.

Polymerase γ is involved in mtDNA synthesis and its integrity. Therefore, we examined if REV3 is involved in maintenance of mtDNA integrity. Rev3^+/+^ MEFs were exposed to UV (5 J/m^2^), and mRNA expression of *Rev3* was examined after 6 and 12 h of UV exposure. In UV-exposed Rev3^+/+^ MEFs but not in unexposed MEFs (data not shown), there was a time-dependent increase in the mRNA expression of *Rev3* (Fig 4A). The mRNA expression status of *Polg1* and *Polg2* in Rev3^+/+^ and Rev3^-/-^ cells was also examined by RT PCR. There was higher expression of both *Polg1* and *Polg2* in Rev3^-/-^ cells relative to Rev3^+/+^ cells (Fig 4B). To confirm the binding of REV3 to mtDNA, chromatin immunoprecipitation (ChIP) assays were accomplished with Rev3^+/+^ cells exposed and not exposed to UV (Fig 4C). PCR amplification of immunoprecipitated mtDNA from two different regions, the D loop (non-coding regulatory region) and COX II (coding region), indicated binding of REV3 to mtDNA (Fig 4C). PCR amplification of both COX II and D loop region in both UV-exposed and unexposed cells indicate that REV3 binds to mtDNA.

To further elucidate the functional importance of REV3 in mitochondria, mtDNA damage was measured after 6 h of UV exposure of cells expressing full-length REV3 or REV3-MLS (REV3 without MLS). There was more mtDNA damage in cells in which REV3 does not localize to mitochondria relative to cells expressing full-length REV3 (Fig 4D). The mtDNA content in Rev3^+/+^ and Rev3^-/-^ MEFs with and without UV exposure was also measured. The mtDNA content recovered in Rev3^+/+^ cells after 6 h of UV exposure (5 J/m^2^) and further increased after 24 h of exposure, whereas, in Rev3^-/-^ cells, UV exposure decreased the mtDNA content compared with unexposed Rev3^-/-^ cells after 6 h; the levels either remained unchanged or only slightly recovered after 24 h of exposure (Fig 4E).

### REV3 is over-expressed in primary human breast tumors and breast cancer cell lines

An imbalance in the expression of polymerase zeta, due either to a decrease in expression or over-expression, is associated with increased genomic instability [37,38]. Thus, a balance in the expression of Pol zeta is necessary for genomic integrity. Although REV3 is involved in genomic stability, its role in human cancers is unclear [9,39–41].

**Fig 5:**
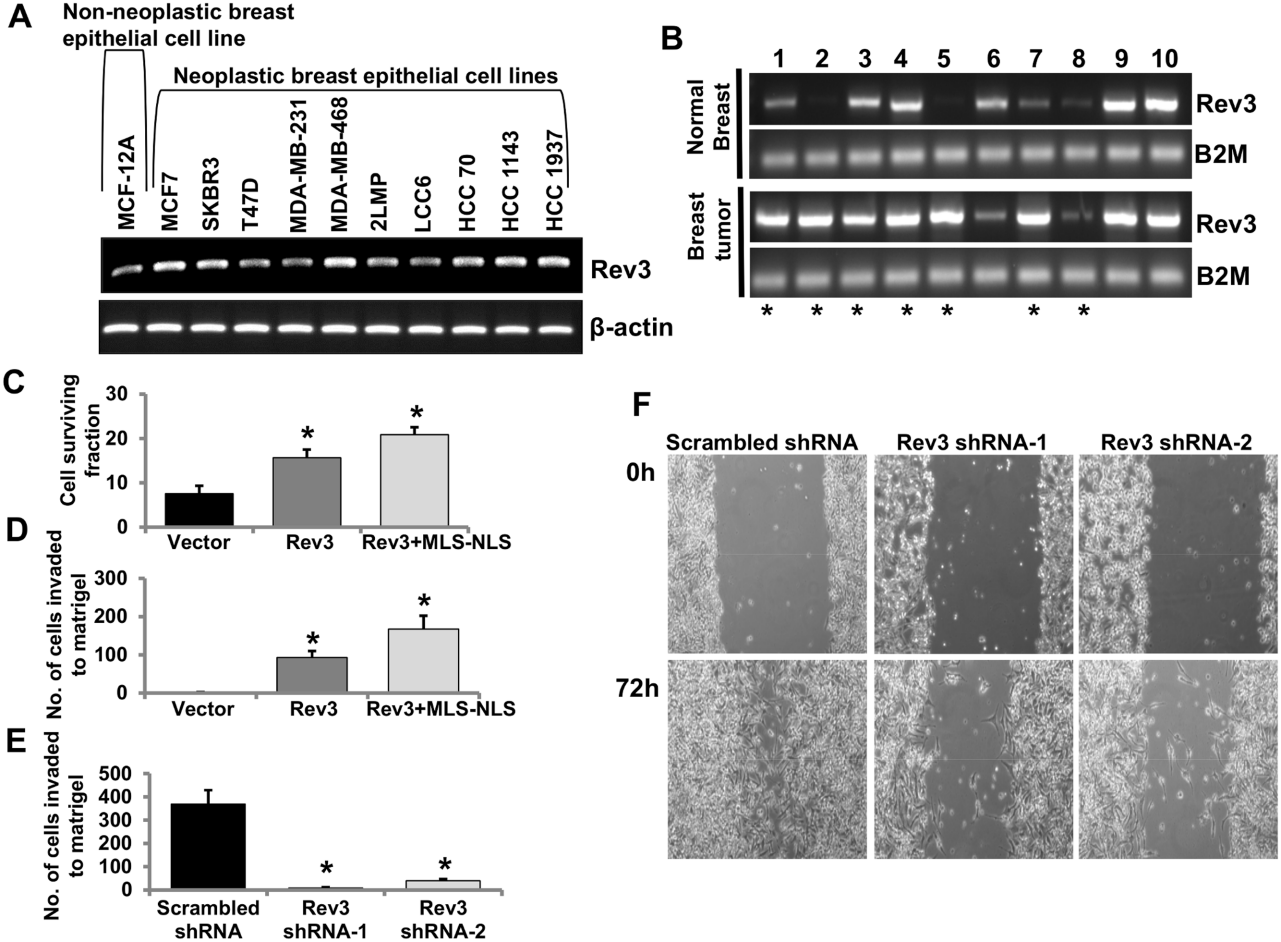
Mitochondrial REV3 contributes to tumorigenic properties. (**A**-**B**) RT PCR analysis of *Rev3* gene expression in the non-neoplastic breast epithelial cell line MCF-12A and in 10 neoplastic breast epithelial cell lines (**A**), and in a set of 10 primary breast tumors and matched normal breast tissues from the same patients (**B**). Breast tumor samples showing increased mRNA expression of *Rev3* compared to respective matched normal breast tissue are denoted by asterisks (*) (**B**). (**C**-**D**) Levels of survival (**C**) and Matrigel invasion capacity (**D**) of full-length *Rev3* and Rev3+MLS-NLS expressing HEK293 cells. Bars represent mean ± s.d. *P<0.05 versus vector-transfected cells; Student's t-test. (**E**) Matrigel invasion capacity of Rev3 shRNA-transfected HeLa cells (Rev3 shRNA-1 and Rev3 shRNA-2). Bars represent the mean ± s.d. *P<0.05 versus scrambled shRNA-transfected HeLa cells; Student's t-test. (**F**) Comparison of cell-culture migration capacity of Rev3 shRNA-transfected HeLa cells (Rev3 shRNA-1 and Rev3 shRNA-2) with scrambled shRNA-transfected HeLa cells.

To assess the expression status of the *Rev3* in human breast cancer, expression of *Rev3* in the non-neoplastic human breast epithelial cell line, MCF-12A, and in 10 human breast epithelial cancer cell lines was determined by RT PCR (Fig 5A). In most of the breast cancer cell lines, there was higher expression of *Rev3* relative to that in MCF-12A cells (Fig 5A). mRNA expression of *Rev3* was also measured in 10 sets of primary breast tumors and matched normal breast tissues derived from the same patients (Fig 5B). Of 10 cases, *Rev3* mRNA expression was higher in 7 breast tumor samples, similar in 2 samples and was lower in one sample relative to their normal breast tissue counterparts (Fig 5A).

### Mitochondrial REV3 contributes to tumorigenic properties

Human REV3 contains both an NLS and an MLS and is present in cytoplasm, nuclei, and mitochondria (Fig 1). To define the role of mitochondrial REV3 in tumorigenesis, full-length *Rev3* or *Rev3* with MLS but without NLS (Rev3+MLS-NLS) were expressed in HEK293 cells (cells with a very low level of endogenous *Rev3* expression). The effect of an increase in expression of full-length REV3 or mitochondrial REV3 on cell survival was determined. HEK293 cells expressing full-length *Rev3* and HEK293 cells expressing Rev3+MLS-NLS were exposed to 7.5 J/m^2^ of UV, as described in the Materials and Methods. After that 2000 UV-exposed cells were plated and allowed to form clones for 10 days, the survival fractions were calculated. Increased expression of *Rev3* or Rev3+MLS-NLS increased survival of HEK293 cells after UV exposure (Fig 5C). Increased expression of *Rev3* or Rev3+MLS-NLS also increased Matrigel invasion of HEK293 cells (Fig 5D).

*Rev3* in HeLa cells (cells with a detectable endogenous level of *Rev3*) was inactivated with two shRNAs specific to *Rev3*. These *Rev3*-knockdown cells were used for assays of Matrigel invasion and cell-culture migration. Lower invasion (Fig 5E) and migration (Fig 5F) after shRNA-mediated knockdown of *Rev3* support a tumorigenic role of *Rev3*.

## Discussion

Present study identified for the first time that human REV3, the catalytic subunit of DNA polymerase zeta localizes to mitochondria and affect mtDNA metabolism. Using MitoProt II software, we identified a mitochondrial localization signal (MLS) at the N-terminal of REV3 protein that helps in translocation of this protein to mitochondria. We analyzed two known isoforms of REV3 synthesized from the same *Rev3* gene via the use of two ATI sites. Presence of ATI sites is one of the gene regulatory mechanisms that diversifies the mammalian proteome and has earlier been reported to be associated with generation of N-terminal protein variants destined to locate to different compartments of the cell [33,42,43]. Use of ATI site to expose a cryptic MLS, located within the coding sequence has earlier been reported for other genes [33]. On the basis of our analyses, we cloned the corresponding cDNA sequence to 107 amino acid long N-terminal region from short isoform of *Rev3* into GFP containing vector and confirmed the ability of this MLS in translocation of GFP into mitochondrial matrix by fluorescent microscopy (Fig 1B), and by western blot with mitochondrial protein fractions (Fig 1C).

In our previous report, we cloned a sequence corresponding to the N-terminal 1–100 amino acids of human REV3 in frame with GFP in the pEGFP-N2 plasmid and evaluated the capacity of this N-terminal region of REV3 for localization of GFP to mitochondria [26], but we were not able to demonstrate the localization of human REV3 into mitochondria. In that study, the putative MLS was identified from the longer isoform of human REV3 [26]. The longer isoform of human REV3 (NP_002903.3) contains extra 78 amino acid long sequence at the N-terminal before the MLS sequence (Fig 1A). Thus, the first 100 amino acid sequence from the longer isoform of human REV3, used in our previous study, lacked an active MLS [26]. Our intense analyses about the active MLS in human REV3 in the present study, confirmed the presence of an active MLS only in the shorter isoform of REV3 synthesized from an ATI site (Fig 1A). Our immunofluorescence analysis of REV3 confirmed localization of full length REV3 in mitochondria (S1 Fig). Western blot analyses of cytoplasmic, nuclear, and mitochondrial fractions from mouse heart as well as human cells indicate that both long (~352 kDa) and short (~343 kDa) isoforms of REV3 are present in the cytoplasm and nuclear fractions while only short isoform of REV3 localizes to mitochondria (Fig 1D). How the presence of an additional 78 amino acid sequence at the N-terminal of the long isoform of human REV3 affects the translocation of this isoform to mitochondria is not yet known.

Our analyses revealed presence of nuclear localization signal (NLS) in both isoforms of REV3 (Fig 1A). We confirmed the ability of this predicted NLS region in translocation of GFP to the nucleus (Fig 1E). To examine effects of REV3 on mitochondrial functions, we used Rev3^+/+^ and Rev3^-/-^ MEFs with p53^-/-^ background [38], and vice versa, i.e., to observe the effects of alterations in mitochondrial OXPHOS on the REV3 expression, we used 143B rho° (mtDNA deficient) cells as well as specific inhibitors of mitochondrial OXPHOS complexes. Decrease in the expression of mitochondrial genome-encoded gene *Cox II*, mtOXPHOS complex IV expression and its enzymatic activity in Rev3^-/-^ cells compared to Rev3^+/+^ cells indicated towards a possible role of REV3 in the maintenance of mitochondrial genome and thus mtOXPHOS functions (Fig 2A, 2B and 2C). Increased glucose consumption rate in Rev3^-/-^ cells (Fig 2D) further points towards compromised OXPHOS system and altered mitochondrial metabolism towards glycolysis, an effect known as Warburg effect [34]. Decreased mitochondrial membrane potential and mitochondrial ROS producing ability in Rev3^-/-^ cells compared to Rev3^+/+^ cells after treatment with CCCP and Antimycin A, respectively, further suggested a role of REV3 in the regulation of mitochondrial functions (Fig 2E and 2F). Increased *Rev3* mRNA expression in 143B cells treated with mitochondrial inhibitors of OXPHOS complexes indicates a compensatory response and also indicates that REV3 might be involved in the maintenance of OXPHOS system (Fig 3A). Increased expression of *Rev3* mRNA in mtDNA deficient 143B rho° cells compared to 143B cells (Fig 3A) again suggests that REV3 expression is linked to mitochondrial function. Overall, these results indicate that REV3 functions in mitochondria and that its inactivation leads to mitochondrial dysfunction.

To date, polymerase γ is the only polymerase described in mitochondria from higher eukaryotes [35,36] but our chromatin immunoprecipitation (ChIP) analyses using mitochondrial fractions from Rev3^+/+^ cells suggest presence of REV3 in mitochondria (Fig 4C). Moreover, association of REV3 with polymerase γ (Fig 3B) demonstrate a cross talk between these two polymerases. Data showing binding of REV3 to two different regions; COX II (coding) and the D loop (non-coding) of mtDNA indicate that REV3 physically interacts with mtDNA irrespective of coding or non-coding region of mtDNA (Fig 4C). Increased binding of REV3 to D loop region of mtDNA, a region known to be more susceptible for DNA damage after UV exposure further indicates towards a possibility of involvement of REV3 in the maintenance of DNA integrity in mitochondria (Fig 4C).

Ultraviolet radiations are known to damage cellular macromolecules. Both nuclear and mtDNA are susceptible to several types of damage caused by UV irradiation [44]. Thymine dimers and 6, 4 photoproducts are the predominant types of damage caused by UV radiation [45]. UV-induced thymine dimers have been shown to distort the nuclear and mtDNA backbone [36,44]. While nuclear DNA damage gets repaired due to presence of a battery of DNA repair enzymes including TLS DNA polymerase, mitochondria are known to lack nuclear-encoded DNA repair enzymes that can repair UV-induced mtDNA damage and thus inherently become more prone to the accumulation of mtDNA damage. Although presence of nuclear encoded base excision repair enzymes in mitochondria has been reported [46,47], yet there is clear lack of evidence that can support presence of TLS DNA polymerase activity in mammalian mitochondria. Lack of polymerase zeta has been shown to confer extreme UV sensitivity [12]. REV3 is not only required to function as DNA damage tolerance system but has also been shown to be required for maintaining genomic integrity and proliferation in normally proliferating cells [12,21].

Increased expression of *Rev3* after UV exposure (Fig 4A) and recovery of mtDNA content only in Rev3^+/+^ cells but not in Rev3^-/-^ cells indicates towards the involvement of REV3 in the maintenance of mtDNA after UV exposure (Fig 4E). Increased mRNA expression of *Polg1* and *Polg2* in the absence of *Rev3* (Fig 4B) and similarly increased mRNA and protein expression of REV3 in presence of a dominant negative POLG1 allele (Fig 3C and 3D) suggest that both polymerases (polymerase γ and REV3) compensate functions of each other in mitochondria and like polymerase γ, REV3 might also be involved in the maintenance of mtDNA (Fig 4D and 4E). A recent report in yeast system also support our results that Pol zeta reduces mitochondrial mutability caused by pathological mutations in *Polg1* gene [48].

Recent evidences support that REV3 alters oncogenic potential of the cells [9,39–41]. Studies have shown effects of REV3 depletion on the cancer cell growth *in vitro* [19,40]. However, dilemma still exists whether REV3 is oncogenic or tumor suppressor in nature. To examine whether *Rev3* behaves as an oncogene or tumor suppressor gene, we cloned and over expressed full length *Rev3* as well as Rev3 containing MLS but without NLS (Rev3+MLS-NLS). Greater cell survival and invasion in case of expression of Rev3+MLS-NLS indicate that mitochondrial REV3 is predominantly responsible for the higher tumorigenicity of the cells (Fig 5C and 5D).

Decreased matrigel invasion as well as cell migration capacity of the cells after shRNA-mediated knocked down of *Rev3* further strengthen the observations that support the role of *Rev3* in development of cancer (Fig 5E and 5F). Association of inhibition of human *Rev3* with less mutagenic properties has been shown earlier [49,50]. Our results indicate that REV3 protects against mtDNA damage (Fig 4D), that its inactivation leads to the Warburg effect (Fig 2D *)*, and that higher mitochondrial expression is associated with increased tumorigenicity of the cells (Fig 5C and 5D).

Increased expression of *Rev3* in human breast epithelial cancer cell lines compared to non-neoplastic human breast epithelial cells MCF-12A, and in human breast tumors compared to normal breast tissues further qualifies *Rev3* as a gene associated with tumorigenesis (Fig 5A and 5B). DNA repair and cell cycle checkpoint mechanisms are frequently abrogated in cancer cells and extent of endogenous DNA damage is higher in tumor tissues [51]. REV3 is a TLS enzyme and helps in maintaining genomic integrity but TLS is a mutagenic process as it often incorporates incorrect nucleotides [52,53]. Thus increased expression of *Rev3* in breast cancer cells and tumor tissues might help carcinogenic processes in two ways, either by acting as a protective mechanism against DNA damage that provide survival advantage to the tumor cells by keeping DNA damage lower than the threshold and/or by inducing mutagenesis during translesion DNA synthesis. Therefore, a balance in the expression of REV3 is required to maintain cellular homeostasis. Overall, our results provide evidences that mammalian REV3 localizes to mitochondria and maintain the integrity of mitochondrial genome. REV3 contributes to tumorigenic potential of cells, however, further experiments are needed to evaluate whether nuclear and mitochondrial REV3 have different effects on tumorigenic activity.

## Supporting Information

S1 Fig Immunofluorescence analysis to show the localization of full length REV3 protein in HEK293 cells. A construct containing full length *Rev3* and a Flag tag (a gift from Dr. Yoshiki Murakumo) was transfected and anti Flag-M2 antibody was used to detect the localization of full length REV3 in mitochondria.
(TIF)

S2 Fig (**A**) Western blot with protein samples from REV3^+/+^ and REV3^-/-^ cells to show the specificity of REV3 antibody (Santa Cruz Biotechnology, Cat # sc-48814) used in this study. (**B**) Western blot showing specificity of REV3 antibody (Santa Cruz Biotechnology, Cat # sc-48814) as well as Rev3 shRNA-mediated knockdown of REV3 in HeLa cells.
(TIF)

## References

[1] Brandon M, Baldi P, Wallace DC (2006) Mitochondrial mutations in cancer. Oncogene 25: 4647–4662. 16892079 10.1038/sj.onc.1209607

[2] Kulawiec M, Safina A, Desouki MM, Still I, Matsui S, Bakin A, et al (2008) Tumorigenic transformation of human breast epithelial cells induced by mitochondrial DNA depletion. Cancer Biol Ther 7: 1732–1743. 19151587 10.4161/cbt.7.11.6729 PMC2783327

[3] Modica-Napolitano JS, Kulawiec M, Singh KK (2007) Mitochondria and human cancer. Curr Mol Med 7: 121–131. 17311537 10.2174/156652407779940495

[4] Warburg O (1956) On respiratory impairment in cancer cells. Science 124: 269–270. 13351639

[5] Copeland WC, Longley MJ (2003) DNA polymerase gamma in mitochondrial DNA replication and repair. ScientificWorldJournal 3: 34–44. 12806118 10.1100/tsw.2003.09 PMC5974855

[6] Kaguni LS (2004) DNA polymerase gamma, the mitochondrial replicase. Annu Rev Biochem 73: 293–320. 15189144 10.1146/annurev.biochem.72.121801.161455

[7] Singh KK, Ayyasamy V, Owens KM, Koul MS, Vujcic M (2009) Mutations in mitochondrial DNA polymerase-gamma promote breast tumorigenesis. J Hum Genet 54: 516–524. 10.1038/jhg.2009.71 19629138 PMC2782392

[8] Copeland WC, Ponamarev MV, Nguyen D, Kunkel TA, Longley MJ (2003) Mutations in DNA polymerase gamma cause error prone DNA synthesis in human mitochondrial disorders. Acta Biochim Pol 50: 155–167. 12673356

[9] Knobel PA, Marti TM (2011) Translesion DNA synthesis in the context of cancer research. Cancer Cell Int 11: 39 10.1186/1475-2867-11-39 22047021 PMC3224763

[10] Waters LS, Minesinger BK, Wiltrout ME, D'Souza S, Woodruff RV, Walker GC (2009) Eukaryotic translesion polymerases and their roles and regulation in DNA damage tolerance. Microbiol Mol Biol Rev 73: 134–154. 10.1128/MMBR.00034-08 19258535 PMC2650891

[11] Nelson JR, Lawrence CW, Hinkle DC (1996) Thymine-thymine dimer bypass by yeast DNA polymerase zeta. Science 272: 1646–1649. 8658138 10.1126/science.272.5268.1646

[12] Lange SS, Bedford E, Reh S, Wittschieben JP, Carbajal S, Kusewitt DF, et al (2013) Dual role for mammalian DNA polymerase zeta in maintaining genome stability and proliferative responses. Proc Natl Acad Sci U S A 110: E687–696. 10.1073/pnas.1217425110 23386725 PMC3581960

[13] Shachar S, Ziv O, Avkin S, Adar S, Wittschieben J, Reissner T, et al (2009) Two-polymerase mechanisms dictate error-free and error-prone translesion DNA synthesis in mammals. EMBO J 28: 383–393. 10.1038/emboj.2008.281 19153606 PMC2646147

[14] Yoon JH, Prakash L, Prakash S (2010) Error-free replicative bypass of (6–4) photoproducts by DNA polymerase zeta in mouse and human cells. Genes Dev 24: 123–128. 10.1101/gad.1872810 20080950 PMC2807347

[15] Bemark M, Khamlichi AA, Davies SL, Neuberger MS (2000) Disruption of mouse polymerase zeta (Rev3) leads to embryonic lethality and impairs blastocyst development in vitro. Curr Biol 10: 1213–1216. 11050391 10.1016/s0960-9822(00)00724-7

[16] Wittschieben J, Shivji MK, Lalani E, Jacobs MA, Marini F, Gearhart PJ, et al (2000) Disruption of the developmentally regulated Rev3l gene causes embryonic lethality. Curr Biol 10: 1217–1220. 11050392 10.1016/s0960-9822(00)00725-9

[17] Rajpal DK, Wu X, Wang Z (2000) Alteration of ultraviolet-induced mutagenesis in yeast through molecular modulation of the REV3 and REV7 gene expression. Mutat Res 461: 133–143. 11018586 10.1016/s0921-8777(00)00047-1

[18] Bhat A, Andersen PL, Qin Z, Xiao W (2013) Rev3, the catalytic subunit of Polzeta, is required for maintaining fragile site stability in human cells. Nucleic Acids Res 41: 2328–2339. 10.1093/nar/gks1442 23303771 PMC3575803

[19] Knobel PA, Kotov IN, Felley-Bosco E, Stahel RA, Marti TM (2011) Inhibition of REV3 expression induces persistent DNA damage and growth arrest in cancer cells. Neoplasia 13: 961–970. 22028621 10.1593/neo.11828 PMC3201572

[20] Van Sloun PP, Varlet I, Sonneveld E, Boei JJ, Romeijn RJ, Eeken JC, et al (2002) Involvement of mouse Rev3 in tolerance of endogenous and exogenous DNA damage. Mol Cell Biol 22: 2159–2169. 11884603 10.1128/MCB.22.7.2159-2169.2002 PMC133679

[21] Lange SS, Wittschieben JP, Wood RD (2012) DNA polymerase zeta is required for proliferation of normal mammalian cells. Nucleic Acids Res 40: 4473–4482. 10.1093/nar/gks054 22319213 PMC3378892

[22] Varadi V, Bevier M, Grzybowska E, Johansson R, Enquist K, Henriksson R, et al (2011) Genetic variation in genes encoding for polymerase zeta subunits associates with breast cancer risk, tumour characteristics and survival. Breast Cancer Res Treat 129: 235–245. 10.1007/s10549-011-1460-z 21455670

[23] Wang H, Zhang SY, Wang S, Lu J, Wu W, Weng L, et al (2009) REV3L confers chemoresistance to cisplatin in human gliomas: the potential of its RNAi for synergistic therapy. Neuro Oncol 11: 790–802. 10.1215/15228517-2009-015 19289490 PMC2802399

[24] Doles J, Oliver TG, Cameron ER, Hsu G, Jacks T, Walker GC, et al (2010) Suppression of Rev3, the catalytic subunit of Pol{zeta}, sensitizes drug-resistant lung tumors to chemotherapy. Proc Natl Acad Sci U S A 107: 20786–20791. 10.1073/pnas.1011409107 21068376 PMC2996428

[25] Xie K, Doles J, Hemann MT, Walker GC (2010) Error-prone translesion synthesis mediates acquired chemoresistance. Proc Natl Acad Sci U S A 107: 20792–20797. 10.1073/pnas.1011412107 21068378 PMC2996453

[26] Zhang H, Chatterjee A, Singh KK (2006) Saccharomyces cerevisiae polymerase zeta functions in mitochondria. Genetics 172: 2683–2688. 16452144 10.1534/genetics.105.051029 PMC1456388

[27] Lin X, Trang J, Okuda T, Howell SB (2006) DNA polymerase zeta accounts for the reduced cytotoxicity and enhanced mutagenicity of cisplatin in human colon carcinoma cells that have lost DNA mismatch repair. Clin Cancer Res 12: 563–568. 16428501 10.1158/1078-0432.CCR-05-1380

[28] Birch-Machin MA, Briggs HL, Saborido AA, Bindoff LA, Turnbull DM (1994) An evaluation of the measurement of the activities of complexes I-IV in the respiratory chain of human skeletal muscle mitochondria. Biochem Med Metab Biol 51: 35–42. 8192914 10.1006/bmmb.1994.1004

[29] O'Malley Y, Fink BD, Ross NC, Prisinzano TE, Sivitz WI (2006) Reactive oxygen and targeted antioxidant administration in endothelial cell mitochondria. J Biol Chem 281: 39766–39775. 17060316 10.1074/jbc.M608268200

[30] Graham KA, Kulawiec M, Owens KM, Li X, Desouki MM, Chandra D, et al (2010) NADPH oxidase 4 is an oncoprotein localized to mitochondria. Cancer Biol Ther 10: 223–231. 20523116 10.4161/cbt.10.3.12207 PMC3040835

[31] Singh B, Bhat HK (2012) Superoxide dismutase 3 is induced by antioxidants, inhibits oxidative DNA damage and is associated with inhibition of estrogen-induced breast cancer. Carcinogenesis 33: 2601–2610. 10.1093/carcin/bgs300 23027624 PMC3510741

[32] Rothfuss O, Gasser T, Patenge N (2010) Analysis of differential DNA damage in the mitochondrial genome employing a semi-long run real-time PCR approach. Nucleic Acids Res 38: e24 10.1093/nar/gkp1082 19966269 PMC2831309

[33] Kazak L, Reyes A, He J, Wood SR, Brea-Calvo G, Holen TT, et al (2013) A cryptic targeting signal creates a mitochondrial FEN1 isoform with tailed R-Loop binding properties. PLoS One 8: e62340 10.1371/journal.pone.0062340 23675412 PMC3652857

[34] Warburg O (1956) On the origin of cancer cells. Science 123: 309–314. 13298683 10.1126/science.123.3191.309

[35] Foury F (1989) Cloning and sequencing of the nuclear gene MIP1 encoding the catalytic subunit of the yeast mitochondrial DNA polymerase. J Biol Chem 264: 20552–20560. 2684980

[36] Kasiviswanathan R, Longley MJ, Chan SS, Copeland WC (2009) Disease mutations in the human mitochondrial DNA polymerase thumb subdomain impart severe defects in mitochondrial DNA replication. J Biol Chem 284: 19501–19510. 10.1074/jbc.M109.011940 19478085 PMC2740576

[37] Li Z, Zhang H, McManus TP, McCormick JJ, Lawrence CW, Maher VM (2002) hREV3 is essential for error-prone translesion synthesis past UV or benzo[a]pyrene diol epoxide-induced DNA lesions in human fibroblasts. Mutat Res 510: 71–80. 12459444 10.1016/s0027-5107(02)00253-1

[38] Wittschieben JP, Reshmi SC, Gollin SM, Wood RD (2006) Loss of DNA polymerase zeta causes chromosomal instability in mammalian cells. Cancer Res 66: 134–142. 16397225 10.1158/0008-5472.CAN-05-2982

[39] Brondello JM, Pillaire MJ, Rodriguez C, Gourraud PA, Selves J, Cazaux C, et al (2008) Novel evidences for a tumor suppressor role of Rev3, the catalytic subunit of Pol zeta. Oncogene 27: 6093–6101. 10.1038/onc.2008.212 18622427

[40] Gueranger Q, Stary A, Aoufouchi S, Faili A, Sarasin A, Reynaud CA, et al (2008) Role of DNA polymerases eta, iota and zeta in UV resistance and UV-induced mutagenesis in a human cell line. DNA Repair (Amst) 7: 1551–1562. 18586118 10.1016/j.dnarep.2008.05.012

[41] Wittschieben JP, Patil V, Glushets V, Robinson LJ, Kusewitt DF, Wood RD (2010) Loss of DNA polymerase zeta enhances spontaneous tumorigenesis. Cancer Res 70: 2770–2778. 10.1158/0008-5472.CAN-09-4267 20215524 PMC3008348

[42] Lakshmipathy U, Campbell C (1999) The human DNA ligase III gene encodes nuclear and mitochondrial proteins. Mol Cell Biol 19: 3869–3876. 10207110 10.1128/mcb.19.5.3869 PMC84244

[43] Suzuki Y, Holmes JB, Cerritelli SM, Sakhuja K, Minczuk M, Holt IJ, et al (2010) An upstream open reading frame and the context of the two AUG codons affect the abundance of mitochondrial and nuclear RNase H1. Mol Cell Biol 30: 5123–5134. 10.1128/MCB.00619-10 20823270 PMC2953059

[44] Meyer JN, Boyd WA, Azzam GA, Haugen AC, Freedman JH, Van Houten B (2007) Decline of nucleotide excision repair capacity in aging Caenorhabditis elegans. Genome Biol 8: R70 17472752 10.1186/gb-2007-8-5-r70 PMC1929140

[45] Friedberg EC, Aguilera A, Gellert M, Hanawalt PC, Hays JB, Lehmann AR, et al (2006) DNA repair: from molecular mechanism to human disease. DNA Repair (Amst) 5: 986–996. 16955546 10.1016/j.dnarep.2006.05.005

[46] Chatterjee A, Singh KK (2001) Uracil-DNA glycosylase-deficient yeast exhibit a mitochondrial mutator phenotype. Nucleic Acids Res 29: 4935–4940. 11812822 10.1093/nar/29.24.4935 PMC97606

[47] Kang D, Nishida J, Iyama A, Nakabeppu Y, Furuichi M, Fujiwara T, et al (1995) Intracellular localization of 8-oxo-dGTPase in human cells, with special reference to the role of the enzyme in mitochondria. J Biol Chem 270: 14659–14665. 7782328 10.1074/jbc.270.24.14659

[48] Baruffini E, Serafini F, Ferrero I, Lodi T (2012) Overexpression of DNA polymerase zeta reduces the mitochondrial mutability caused by pathological mutations in DNA polymerase gamma in yeast. PLoS One 7: e34322 10.1371/journal.pone.0034322 22470557 PMC3314619

[49] Gibbs PE, McGregor WG, Maher VM, Nisson P, Lawrence CW (1998) A human homolog of the Saccharomyces cerevisiae REV3 gene, which encodes the catalytic subunit of DNA polymerase zeta. Proc Natl Acad Sci U S A 95: 6876–6880. 9618506 10.1073/pnas.95.12.6876 PMC22668

[50] Kozmin SG, Pavlov YI, Kunkel TA, Sage E (2003) Roles of Saccharomyces cerevisiae DNA polymerases Poleta and Polzeta in response to irradiation by simulated sunlight. Nucleic Acids Res 31: 4541–4552. 12888515 10.1093/nar/gkg489 PMC169879

[51] Curtin NJ (2012) DNA repair dysregulation from cancer driver to therapeutic target. Nat Rev Cancer 12: 801–817. 10.1038/nrc3399 23175119

[52] Nair DT, Johnson RE, Prakash L, Prakash S, Aggarwal AK (2005) Rev1 employs a novel mechanism of DNA synthesis using a protein template. Science 309: 2219–2222. 16195463 10.1126/science.1116336

[53] Prakash S, Johnson RE, Prakash L (2005) Eukaryotic translesion synthesis DNA polymerases: specificity of structure and function. Annu Rev Biochem 74: 317–353. 15952890 10.1146/annurev.biochem.74.082803.133250

