# Supplementary figures and images for: Human REV3 DNA Polymerase Zeta Localizes to Mitochondria and Protects the Mitochondrial Genome
Source: PLoS One. 2015 Oct 13;10(10):e0140409. doi: 10.1371/journal.pone.0140409 (PMC4604079; doi:10.1371/journal.pone.0140409)

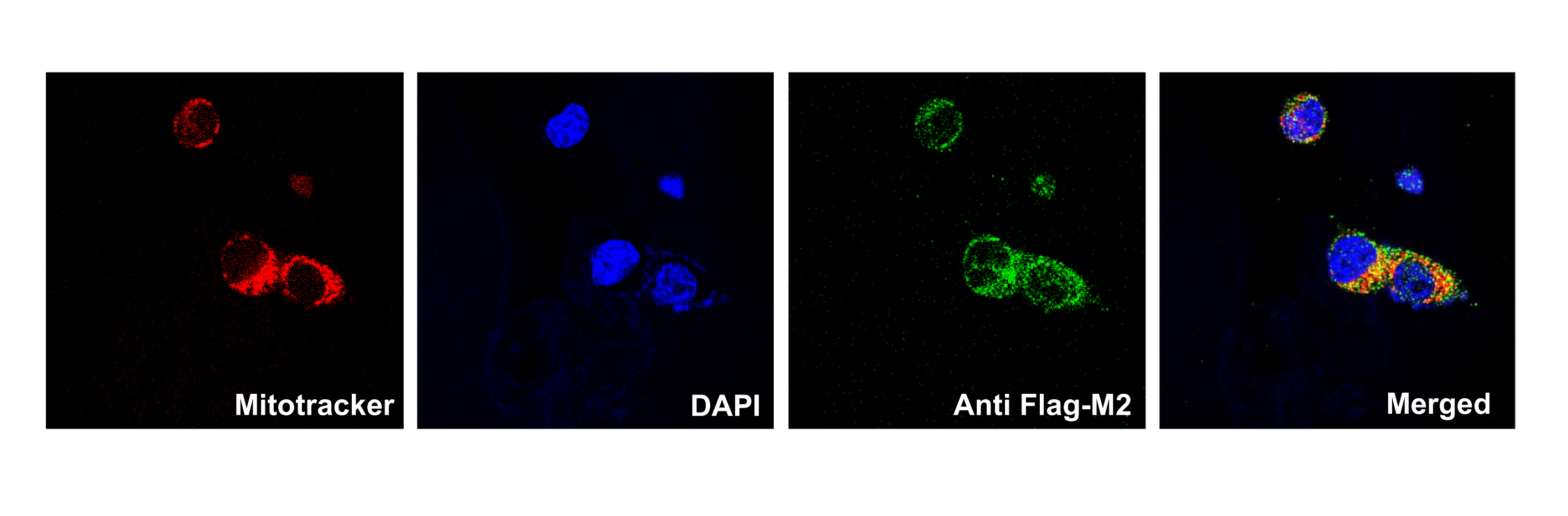

Supplement: S1 Fig — A construct containing full length Rev3 and a Flag tag (a gift from Dr. Yoshiki Murakumo) was transfected and anti Flag-M2 antibody was used to detect the localization of full length REV3 in mitochondria. (TIF) [file pone.0140409.s001.tif]

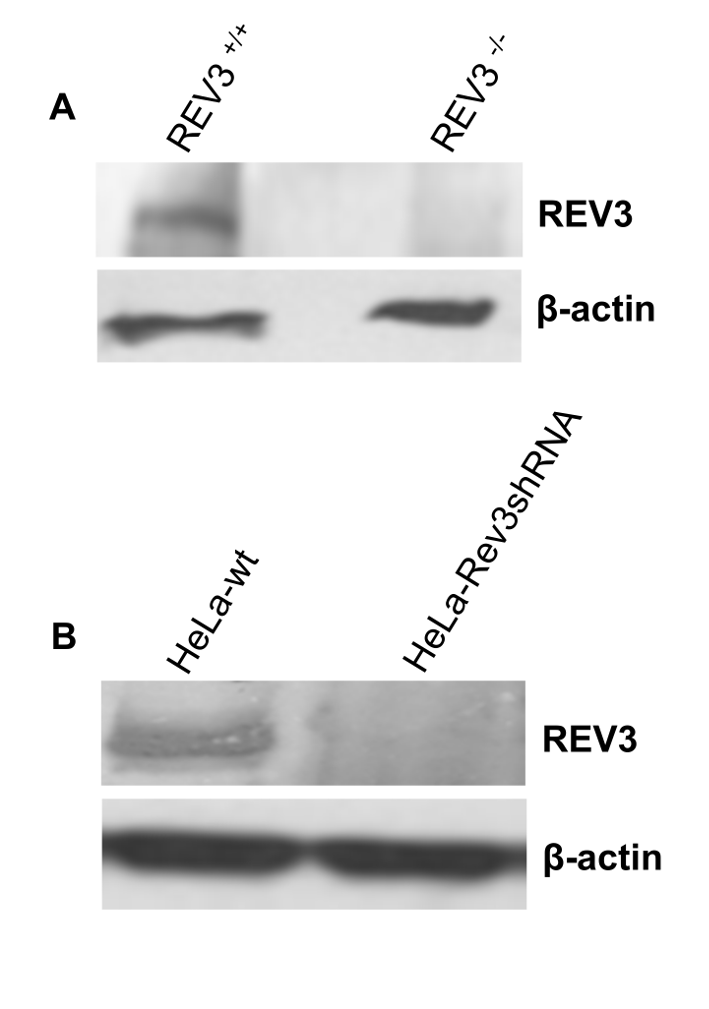

Supplement: S2 Fig — (A) Western blot with protein samples from REV3+/+ and REV3-/- cells to show the specificity of REV3 antibody (Santa Cruz Biotechnology, Cat # sc-48814) used in this study. (B) Western blot showing specificity of REV3 antibody (Santa Cruz Biotechnology, Cat # sc-48814) as well as Rev3 shRNA-mediated knockdown of REV3 in HeLa cells. (TIF) [file pone.0140409.s002.tif]
